# Sleep patterns and impaired cognitive function in adults aged ≥ 55 years: a longitudinal study in China

**DOI:** 10.1186/s12889-026-27979-w

**Published:** 2026-06-02

**Authors:** Wei Zhang, DeQing Zeng, Yuge Yan, YiJing Chu, Yuqi Yang, Jing Guo, Jie Shen, Jiaoling Huang

**Affiliations:** 1 School of Public Health, Shanghai Jiao Tong University, Shanghai, China; 2 Shanghai Institute of Infectious Disease and Biosecurity, Fudan University, Shanghai, China; 3 School of Social Development & Public Policy, Fudan University, Shanghai, China; 4 Medical Records and Statistics Office, Shanghai Sixth People’s Hospital, Shanghai, China

**Keywords:** Sleep pattern, Dementia, AACD-defined cognitive impairment, Older people

## Abstract

**Background:**

Research on sleep duration and cognitive function has primarily relied on cross-sectional designs or single-time assessments, overlooking the longitudinal patterns of sleep behavior. This study aims to explore the synergistic effects of nighttime sleep and daytime napping on cognitive changes.

**Methods:**

We identified distinct long-term sleep patterns using Dynamic Time Warping -based K-medoids clustering analysis of three sleep data sets from 4,385 participants aged 55 and older. We examined the association between these patterns and cognitive decline and AACD-defined cognitive impairment incidence via linear mixed-effects models and time-censored Cox proportional hazards regression analysis.

**Results:**

Three sleep pattern clusters were identified: Cluster 1 (moderate-stable sleep duration, increasing napping), Cluster 2 (long-stable sleep, frequent napping), and Cluster 3 (progressively shorter sleep, infrequent napping). Compared with Cluster 1, both Cluster 2 (β = -0.11, 95% CI: -0.16 to -0.05) and Cluster 3 (β = -0.09, 95% CI: -0.13 to -0.04) exhibited significantly greater cognitive decline. In fully adjusted Cox models, Cluster 3 was modestly associated with a higher likelihood of AACD-defined cognitive impairment (HR = 1.15, 95% CI: 1.02 to 1.23), while Cluster 2 showed no significant association.

**Conclusions:**

Distinct long-term sleep-nap patterns are differentially associated with cognitive outcomes. Shortening sleep duration over time with limited napping may be associated with a higher likelihood of AACD-defined cognitive impairment. These findings highlight the potential importance of monitoring longitudinal sleep behaviors in older adults, although further studies are needed to clarify the underlying mechanisms.

**Supplementary Information:**

The online version contains supplementary material available at 10.1186/s12889-026-27979-w.

**Table:** 

Text box 1. Contributions to the Literature
• Moves beyond cross-sectional and single-time sleep measures by identifying longitudinal sleep–nap trajectories, addressing a key gap in sleep–cognition research.
• Applies DTW-based clustering to characterize dynamic sleep behaviors, advancing methodological approaches in population health studies.
• Provides longitudinal evidence linking multidimensional sleep patterns to cognitive decline and AACD-defined cognitive impairment in older adults.
• Highlights combined nighttime and daytime sleep behaviors as potential targets for monitoring and early public health intervention.

## Background

Dementia represents one of the foremost contributors to global disability-adjusted life year, with increasing prevalence particularly in developing regions [1]. With one new case emerging every 3 s worldwide, current projections estimate 139 million individuals will be living with dementia by 2050 [2]. According to the latest global burden of disease study data, Alzheimer’s disease and other dementias rank ninth among global causes of death [3]. Mild cognitive impairment (MCI) is considered a transitional stage between normal cognitive function and dementia. Early intervention for MCI is beneficial in preventing the onset of dementia [4]. An increasing number of studies have found that the onset age of dementia is shifting earlier. Research indicates that the age-standardized prevalence rate of dementia is 119.0 per 100,000 people [5], underscoring the critical importance of studying early dementia prevention.

Sleep constitutes a modifiable lifestyle factor critically influencing cognitive health, particularly in aging populations [6]. Epidemiological evidence demonstrates a U-shaped association between sleep duration and both cognitive decline and MCI risk [7], indicating that deviations from optimal sleep duration (either excessive or insufficient) significantly increase vulnerability to cognitive impairment. However, null associations have been reported in some studies [8, 9]. Notably, predominant reliance on cross-sectional designs or single-time assessments limits examination of individual sleep trajectory variations and their long-term cognitive consequences. Furthermore, daytime napping represents a prevalent behavioral pattern in older populations. Emerging evidence suggests that moderate napping duration shows potential cognitive benefits whereas prolonged napping is associated with elevated MCI risk [10, 11]. Napping practices are strongly influenced by sociocultural factors, with higher prevalence and more positive health perceptions in Asian populations, especially in China. Most existing research examines nighttime sleep and daytime napping separately, overlooking their combined effects on cognition. In addition, traditional categorical classifications (e.g., short, normal, long) are subjective, and few studies have integrated sleep duration variability with napping patterns in a comprehensive analysis.

To address these limitations, this study used multiple measurements from a large cohort to build time-series models combining nocturnal sleep duration and daytime napping, and applied clustering to identify distinct long-term sleep–nap patterns. We then examined their associations with cognitive decline and AACD-defined cognitive impairment to reveal sleep behavior types and their long-term effects on cognition, which may provide insights for future research on sleep-related cognitive health in older adults.

## Methods

### Study design and participants

The China Health and Retirement Longitudinal Study (CHARLS) is a nationwide survey of Chinese adults aged 45 and older. Initiated in 2011, subsequent waves are conducted every two to three years to assess health, psychological, socioeconomic, and insurance-related indicators [12]. This project has received ethical approval from the Biomedical Ethics Review Committee of Peking University (approval number IRB00001052-11015), and all participants signed written informed consent forms prior to inclusion in the study.

This study was a secondary longitudinal analysis using existing cohort data from the CHARLS. Three waves of CHARLS data (Wave 0 to Wave 2) collected between 2011 and 2015 were included in the present analysis. The baseline survey covered a total of 17,705 participants. Individuals aged 55 years and older were included because sleep-related changes and early cognitive decline may emerge before the conventional thresholds of 60 or 65 years [13, 14]. After screening, ultimately enrolled 4,385 participants with complete measurement data. The detailed participant screening process is described in Figure S1.

### Outcome and sleep patterns

The cognitive subscale in CHARLS covers cognitive function dimensions including orientation, memory, attention and calculation, and drawing ability, with a score range of 0–31 points [15]. Specific questions are detailed in Appendix I. Variable Measurement in CHARLS.

Based on the aforementioned cognitive function scores, we established two primary outcome measures: cognitive decline and AACD-defined cognitive impairment. To compare the rates of cognitive decline across different groups, standardized Z-scores based on baseline scores were calculated. Subsequently, the Z-scores for the four cognitive dimensions were averaged and normalized to baseline to obtain the overall cognitive Z-score. A Z-score of -1 indicates cognitive performance one standard deviation below the baseline mean [7]. The operationalization of MCI has exhibited significant methodological heterogeneity across existing studies. In the present study, cognitive impairment was operationally defined according to aging-associated cognitive decline (AACD) criteria [16, 17]. Participants were stratified into 5-year age cohorts, with those demonstrating cognitive scores ≥ 1 standard deviation (SD) below the age-specific mean being classified as having AACD-defined cognitive impairment, which is consistent with prior epidemiological studies [18].

Sleep parameters, including self-reported nighttime sleep duration and daytime napping frequency, were collected through questionnaires. Participant subgroups with distinct patterns of self-reported nighttime sleep duration and napping frequency were identified using a Dynamic Time Warping (DTW)-based K-medoids clustering algorithm [19, 20] incorporating both sleep parameters and longitudinal temporal patterns across follow-up assessments. Basic demographic variables (age, gender, marital status, education), lifestyle factors (social activities, alcohol consumption, smoking, body mass index (BMI) and health status (systolic blood pressure, diastolic blood pressure, hypertension, diabetes mellitus or hyperglycemia, heart attack, stroke, depression were included as confounders or covariates (Appendix I. Variable Measurement in CHARLS).

### Statistical analysis

The clustering analysis employed a DTW-enhanced K-medoids algorithm integrating longitudinal sleep duration and daytime napping patterns. Longitudinal sleep data were reshaped into individual time series and analyzed using DTW-based partitional clustering with partitioning around medoids as the centroid method. The DTW distance metric was used to measure similarity between sleep trajectories. Cluster analysis was conducted with 100 iterations and a fixed random seed to ensure reproducibility. The optimal number of clusters was determined based on silhouette coefficient analysis and trajectory interpretability, with K = 3 selected as the final solution (silhouette score = 0.300). Principal component analysis (PCA) was used to visualize cluster separation. Baseline characteristics were compared across sleep pattern subgroups using descriptive statistics. Continuous variables were summarized using means and standard deviations, while categorical variables were expressed as counts (percentages). Inter-group differences were assessed using one-way ANOVA for continuous variables and χ² tests for categorical variables.

Longitudinal associations between sleep pattern subtypes and cognitive decline (standardized Z-scores) were evaluated using linear mixed-effects models. The model accounted for repeated cognitive assessments by specifying participant-specific random intercepts while treating time as a fixed effect. Three hierarchical models were specified: (1) an unadjusted model examining crude associations; (2) a demographically-adjusted model; and (3) a fully-adjusted model including lifestyle and health status.

Cox proportional hazards regression with follow-up duration as the underlying time metric was employed to examine associations between sleep pattern phenotypes and AACD-defined cognitive impairment. Because AACD-defined cognitive impairment was assessed only at discrete survey waves, the exact timing of onset could not be determined. Therefore, the Cox proportional hazards model was applied as an approximation of the interval-censored event process. Survival time is defined as the time from the baseline to the first observed occurrence of AACD-defined cognitive impairment or the end of follow-up. Participants with AACD-defined cognitive impairment at baseline were excluded from the survival analyses. Because sleep trajectory patterns were derived from repeated measurements across multiple survey waves, the temporal relationship between sleep trajectories and cognitive outcomes should be interpreted cautiously. The proportional hazards assumption was verified through Schoenfeld residual testing. Mirroring the analytical approach for cognitive decline, three hierarchically-adjusted Cox models were specified with progressive covariate inclusion, accompanied by covariate-adjusted survival function estimation. Multicollinearity was assessed using variance inflation factors (VIFs) and generalized variance inflation factors (GVIFs), and no substantial multicollinearity was detected. Subgroup analysis of the sleep model and AACD-defined cognitive impairment was conducted using the following variables: age (55–59, 60–64, 65–69, 70–74, ≥ 75), gender (male, female), marital status (married, single), educational attainment (elementary school, illiterate, middle school, undergraduate and above), social activities (yes, no), alcohol consumption (< 1 time per month, ≥ 1 time per month, none), smoking (yes, no), and BMI (normal, obesity, overweight, underweight). All statistical analyses were performed using Python version 3.13 and R version 4.4.3, utilizing the R packages ‘dtwclust’ ‘lme4’ and ‘survival’.

## Result

### Sleep pattern clustering

**Fig. 1:**
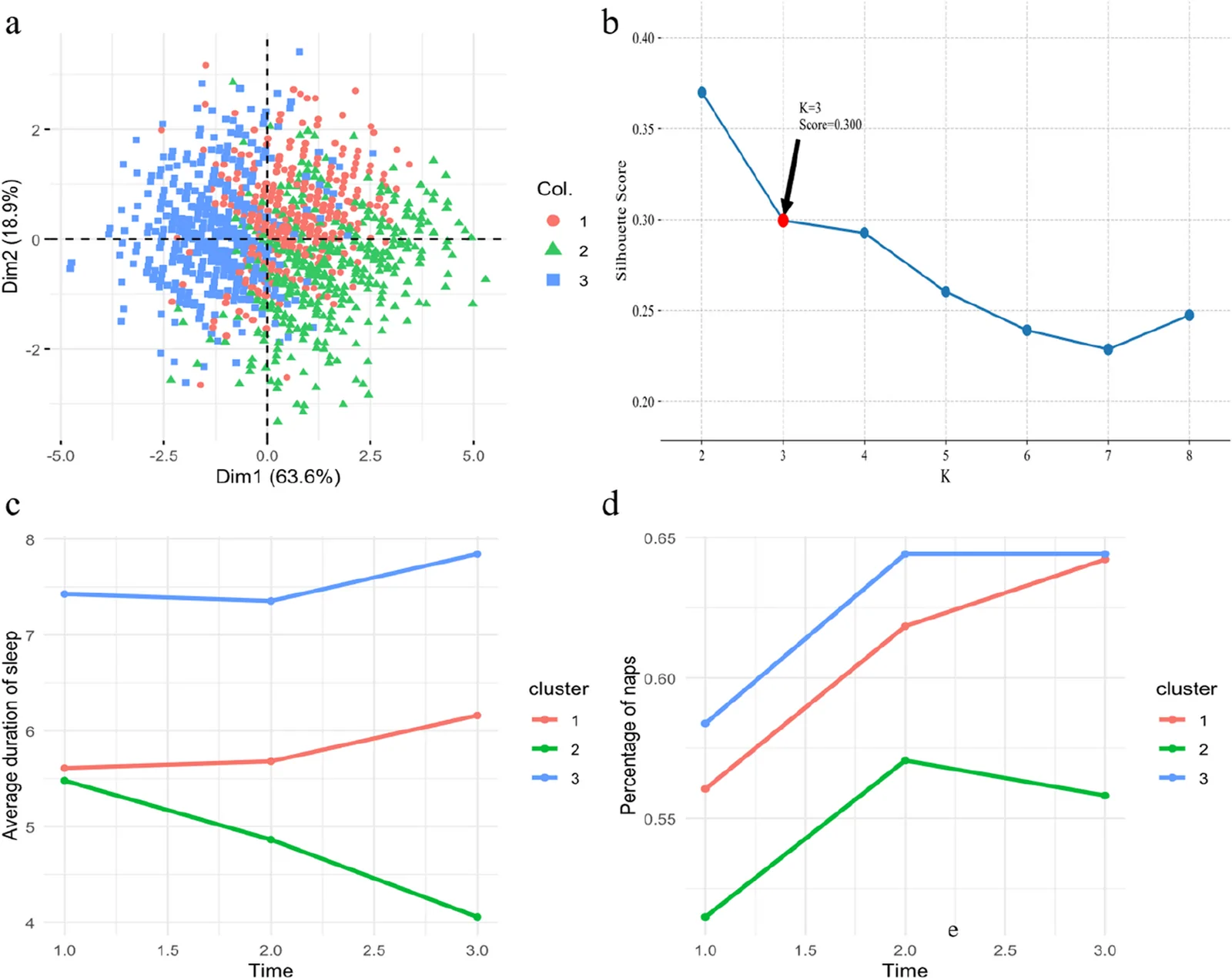
Time series-based DTW and K-medoids clustering solutions using night sleep duration and napping **a** PCA visualization of participant distribution across the three sleep trajectory clusters **b** Silhouette coefficient analysis for determining the optimal number of clusters, with K = 3 selected as the final clustering solution (silhouette score = 0.300) **c** Longitudinal trajectories of average nighttime sleep duration across the three clusters **d** Longitudinal trajectories of daytime napping frequency across the three clusters

K-medoids clustering based on DTW identified 3 distinct sleep trajectory subgroups among participants (silhouette score = 0.30, Fig. 1). Cluster 1 (*n* = 1554) was characterized by moderately stable nighttime sleep duration accompanied by an increasing daytime napping frequency. Cluster 2 (*n* = 1041) exhibited relatively long and stable sleep duration with frequent daytime napping. Cluster 3 (*n* = 1790) showed progressively shorter nighttime sleep duration with relatively infrequent daytime napping.

Panel (a) presents the PCA results illustrating the distribution of participants across the three identified clusters. Longitudinal trends in nighttime sleep duration and daytime napping frequency for each cluster are shown in Fig. 1c and d, respectively.

### Participant Characteristics

Participants were grouped into three categories based on their sleep pattern characteristics using a clustering method. Table 1 describes the characteristics of participants classified by sleep pattern. The final cohort (mean age = 63.0 ± 6.11 years) included 2,580 males (58.8%) and 1,805 females (41.2%). There were significant differences between participants in these three groups in terms of age, gender, education, drinking last year, current smoking, BMI, heart attack and depression. Post hoc analyses revealed significant age differences [F (2, 4382) = 7.47, *p* < 0.001], with participants in Cluster 1 being significantly younger than those in Cluster 2 (*p* = 0.01), and Cluster 3 participants younger than Cluster 2 (*p* < 0.001). Gender, drinking last year, and current smoking status showed significant variation between Cluster 1 and Cluster 2, as well as between Cluster 2 and Cluster 3. Education differed significantly between cluster 1 and cluster 2 (*p* < 0.001). Heart attack differed between cluster 1 and cluster 3 (*p* < 0.01); and depression differed significantly between all groups (*p* < 0.001) (Table S1).

**Table 1:** Characteristics of participants classified by sleep pattern

	Total (*n* = 4385)	Cluster1 (*n* = 1554)	Cluster2 (*n* = 1041)	Cluster3 (*n* = 1790)	*P* value
Basic demographic information					
Age (mean (SD))	63 (6.11)	62.82 (5.98)	63.64 (6.28)	62.78 (6.10)	< 0.01
Gender (%)					
Female	1805 (41.2)	592 (38.1)	546 (52.4)	667 (37.3)	< 0.001
Male	2580 (58.8)	962 (61.9)	495 (47.6)	1123 (62.7)	.
Marriage (%)					
Married	3742 (85.3)	1339 (86.2)	868 (83.4)	1535 (85.8)	0.118
Single	643 (14.7)	215 (13.8)	173 (16.6)	255 (14.2)	.
Education (%)					
Illiteracy	594 (38.2)	476 (45.7)	740 (41.3)	594 (38.2)	< 0.001
Elementary school	467 (30.1)	319 (30.6)	542 (30.3)	467 (30.1)	
Middle school	464 (29.9)	231 (22.2)	485 (27.1)	464 (29.9)	.
Undergraduate and above	29 ( 1.9)	15 ( 1.4)	23 ( 1.3)	29 ( 1.9)	.
Lifestyle information					
Last month social activities (%)					
No	2104 (48.0)	742 (47.7)	508 (48.8)	854 (47.7)	0.833
Yes	2281 (52.0)	812 (52.3)	533 (51.2)	936 (52.3)	.
Drinking last year (%)					
< 1 time a month	345 (7.87)	137 ( 8.8)	62 ( 6.0)	146 ( 8.2)	< 0.001
>=1 time a month	1300 (29.6)	497 (32.0)	267 (25.6)	536 (29.9)	.
None	2740 (62.5)	920 (59.2)	712 (68.4)	1108 (61.9)	.
Current smoking (%)					
No	2796 (63.8)	978 (62.9)	728 (69.9)	1090 (60.9)	< 0.001
Yes	1589 (36.2)	576 (37.1)	313 (30.1)	700 (39.1)	.
BMI (%)					
Normal	2407 (54.9)	871 (56.0)	578 (55.5)	958 (53.5)	0.233
Obesity	451 (10.3)	156 (10.0)	109 (10.5)	186 (10.4)	.
Overweight	1242 (28.3)	433 (27.9)	273 (26.2)	536 (29.9)	.
Underweight	285 (6.5)	94 ( 6.0)	81 ( 7.8)	110 ( 6.1)	.
Health status information					
Systolic Pressure (mean (SD))	131.47 (21.13 )	130.98 (20.32)	132.39 (21.73)	131.35 (21.46)	0.239
Diastolic Pressure (mean (SD))	75.43 (11.93)	75.57 (11.70)	74.87 (11.66)	75.62 (12.28)	0.22
Hypertension (%)					
No	3197 (72.9)	1124 (72.3)	756 (72.6)	1317 (73.6)	0.345
Yes	1188 (27.1)	430 (27.7)	285 (27.4)	473 (26.4)	.
Diabates or High Blood Sugar (%)					
No	4102 (93.6)	1453 (93.5)	972 (93.4)	1677 (93.7)	0.839
Yes	283 (6.45)	101 ( 6.5)	69 ( 6.6)	113 ( 6.3)	.
Heart attack (%)					
No	3809 (86.9)	1340 (86.2)	881 (84.6)	1588 (88.7)	< 0.001
Yes	576 (13.1)	214 (13.8)	160 (15.4)	202 (11.3)	.
Stroke (%)					
No	4292 (97.9)	1521 (97.9)	1016 (97.6)	1755 (98.0)	0.388
Yes	93 (2.12)	33 ( 2.1)	25 ( 2.4)	35 ( 2.0)	.
Depression (%)					
No	1132 (25.8)	422 (27.2)	379 (36.4)	331 (18.5)	< 0.001
Yes	3253 (74.2)	1132 (72.8)	662 (63.6)	1459 (81.5)	.

### Sleep pattern and cognitive function score decline

Table 2 displays the results from three linear mixed-effects models examining the longitudinal association between sleep pattern clusters and cognitive decline, with adjustments for covariates. Compared with Cluster 1 (reference), both Cluster 2 and Cluster 3 were significantly associated with greater decline in total cognitive function score across all models. In the domains of orientation and memory, cluster 2and 3 showed significant associations with cognitive decline. For attention and calculation, only Cluster 2 showed a marginally significant association in Model 3 (β = -0.05, 95% CI: -0.11 to 0.003, *P* = 0.064), while Cluster 3 did not reach statistical significance (*P* > 0.05 in all models). Regarding copying domain, the decline was significant for Cluster 2 (β = -0.06, 95% CI: -0.11 to -0.01, *P* < 0.05) in the fully adjusted model. Cluster 3 showed a non-significant trend toward decline (*P* = 0.089).

**Table 2:** Longitudinal association between sleep pattern and cognitive function score decline

	Model 1*		Model 2**		Model 3***	
	β (95% Cl)	*P* value	β (95% Cl)	*P* value	β (95% Cl)	*P* value
Total score						
Cluster 1	ref	NA	ref	NA	ref	NA
Cluster 2	-0.22 (-0.28 to -0.15)	< 0.001	-0.12 (-0.18 to -0.07)	< 0.001	-0.11 (-0.16 to -0.05)	< 0.001
Cluster 3	-0.09 (-0.15 to -0.04)	< 0.001	-0.07(-0.11 to -0.02)	< 0.01	-0.09 (-0.13 to -0.04)	< 0.001
Orientation						
Cluster 1	ref	NA	ref	NA	ref	NA
Cluster 2	-0.16 (-0.22 to -0.10)	< 0.001	-0.09 (-0.14 to -0.03)	< 0.01	-0.07 (-0.13 to -0.02)	< 0.01
Cluster 3	-0.08 (-0.13 to -0.03)	< 0.01	-0.05 (-0.10 to -0.01)	< 0.05	-0.07 (-0.12 to -0.03)	< 0.01
Memory						
Cluster 1	ref	NA	ref	NA	ref	NA
Cluster 2	-0.16 (-0.22 to -0.01)	< 0.001	-0.18 (-0.16 to -0.05)	< 0.001	-0.09 (-0.14 to -0.04)	< 0.001
Cluster 3	-0.07 (-0.12 to -0.02)	< 0.01	-0.05 (-0.10 to -0.003)	< 0.05	-0.07 (-0.11 to -0.02)	< 0.01
Attention and Calculation						
Cluster 1	ref	NA	ref	NA	ref	NA
Cluster 2	-0.14 (-0.20 to -0.08)	< 0.001	-0.06 (-0.12 to -0.01)	< 0.05	-0.05 (-0.11 to 0.003)	0.064
Cluster 3	-0.05 (-0.10 to 0.003)	0.067	-0.03 (-0.08 to 0.012)	0.19	-0.04 (-0.09 to 0.003)	0.07
Copying						
Cluster 1	ref	NA	ref	NA	ref	NA
Cluster 2	-0.17 (-0.23 to -0.11)	< 0.001	-0.08 (-0.13 to -0.02)	< 0.01	-0.06 (-0.11 to -0.01)	< 0.05
Cluster 3	-0.05 (-0.01 to 0.003)	0.063	-0.03 (-0.07 to 0.02)	0.25	-0.04 (-0.08 to 0.01)	0.089

*Model 1 is a crude model of cognitive decline and cluster

**Model 2 adjusts for age, gender, marriage, education, and BMI

***Model 3 additionally adjusted for systolic pressure, diastolic pressure, hypertension, diabetes or high blood sugar, heart attack, stroke, depression, last month’s social activities, alcohol consumption in the past year, current smoking status and BMI

### Sleep patterns and the risk of AACD-defined cognitive impairment

**Fig. 2:**
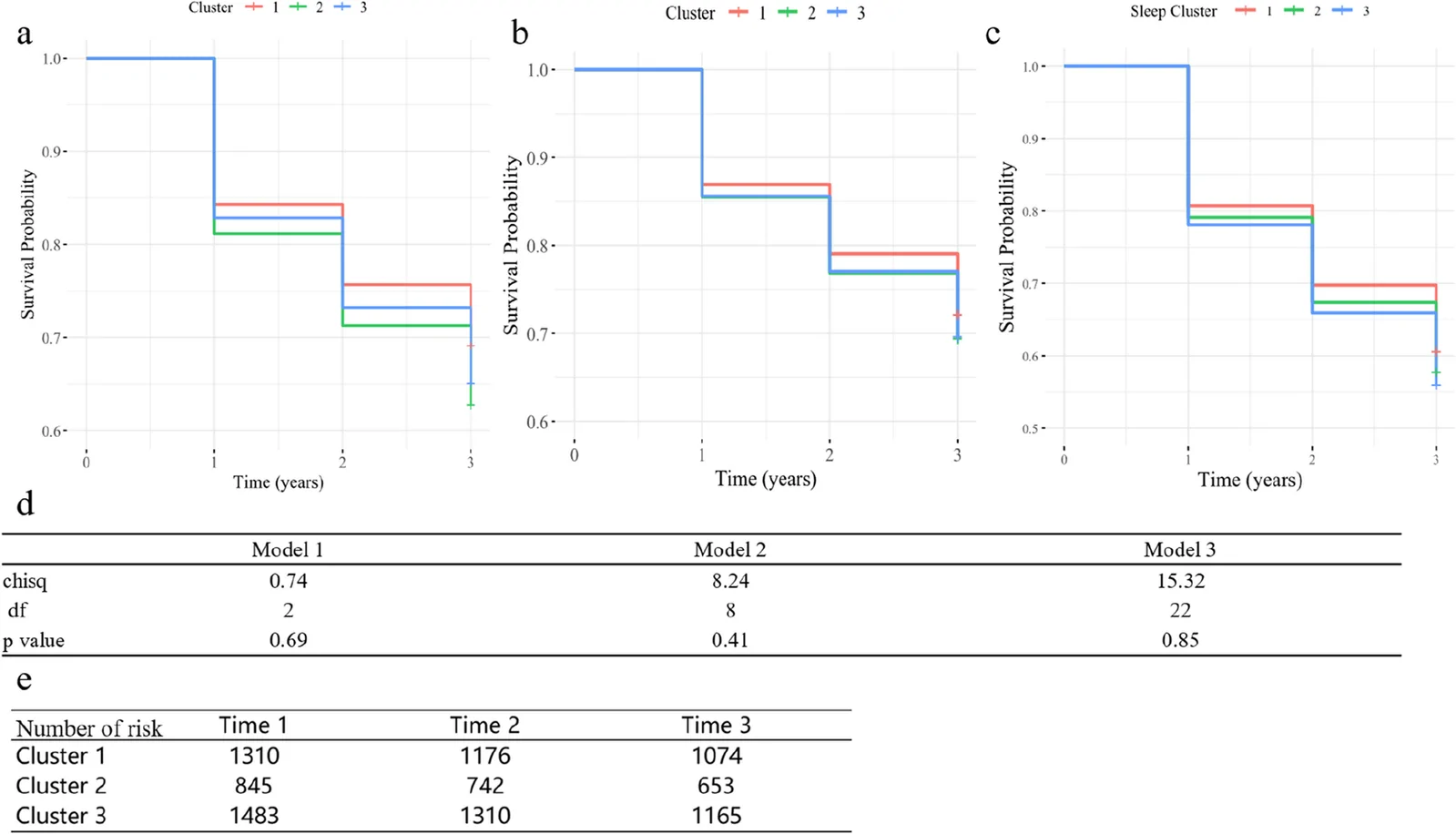
Adjusted survival curves for AACD-defined cognitive impairment by sleep clusters, based on Cox proportional hazards models **a** Model 1 is a crude model of AACD-defined cognitive impairment and cluster **b** Model 2 adjusts for age, gender, marriage, and education **c** Model 3 additionally adjusted for systolic pressure, diastolic pressure, hypertension, diabetes or high blood sugar, heart attack, stroke, depression, last month's social activities, alcohol consumption in the past year, current smoking status and BMI **d** Test of the proportional hazards assumption across three Cox regression models using Schoenfeld residuals (chisq: chi-squared statistic; df: degrees of freedom) **e** Number at risk for each cluster over time

During follow-up, a total of 1,493 participants met the criteria for AACD-defined cognitive impairment, accounting for 34.05% of the study cohort. The cumulative occurrence of AACD-defined cognitive impairment differed across sleep pattern clusters, with Cluster 2 exhibiting the highest incidence (22.72%), followed by Cluster 3 (21.44%), while Cluster 1 had the lowest incidence (18.02%). We constructed three Cox proportional hazards regression models to evaluate the relationship between sleep pattern clusters and AACD-defined cognitive impairment (Table S2). Figure 2 presents the Kaplan-Meier survival curves depicting time to the first observed occurrence of AACD-defined cognitive impairment across the three sleep pattern clusters. In the unadjusted Model 1, participants in Cluster 2 and Cluster 3 showed significantly higher risks of AACD-defined cognitive impairment compared to Cluster 1. These patterns were generally consistent with the separation observed in the Kaplan–Meier survival curves(Fig. 2, a). After adjustment in Model 2, the associations attenuated and lost statistical significance. Nevertheless, the adjusted survival curves maintained partial separation across clusters (Fig. 2, b). In the fully adjusted Model 3, Cluster 3 was modestly associated with a higher likelihood of AACD-defined cognitive impairment (HR = 1.15, 95% CI: 1.02–1.23, *P* < 0.05), whereas the association for Cluster 2 was further attenuated (HR = 1.10, 95% CI: 0.95–1.23, *P* = 0.19). This difference between Cluster 3 and Cluster 1 was also reflected in the adjusted Kaplan–Meier curves (Fig. 2, c). All Cox proportional hazards models satisfied the proportional hazards assumption (Fig. 2, d, *p* > 0.05).

### Subgroup analysis

**Fig. 3:**
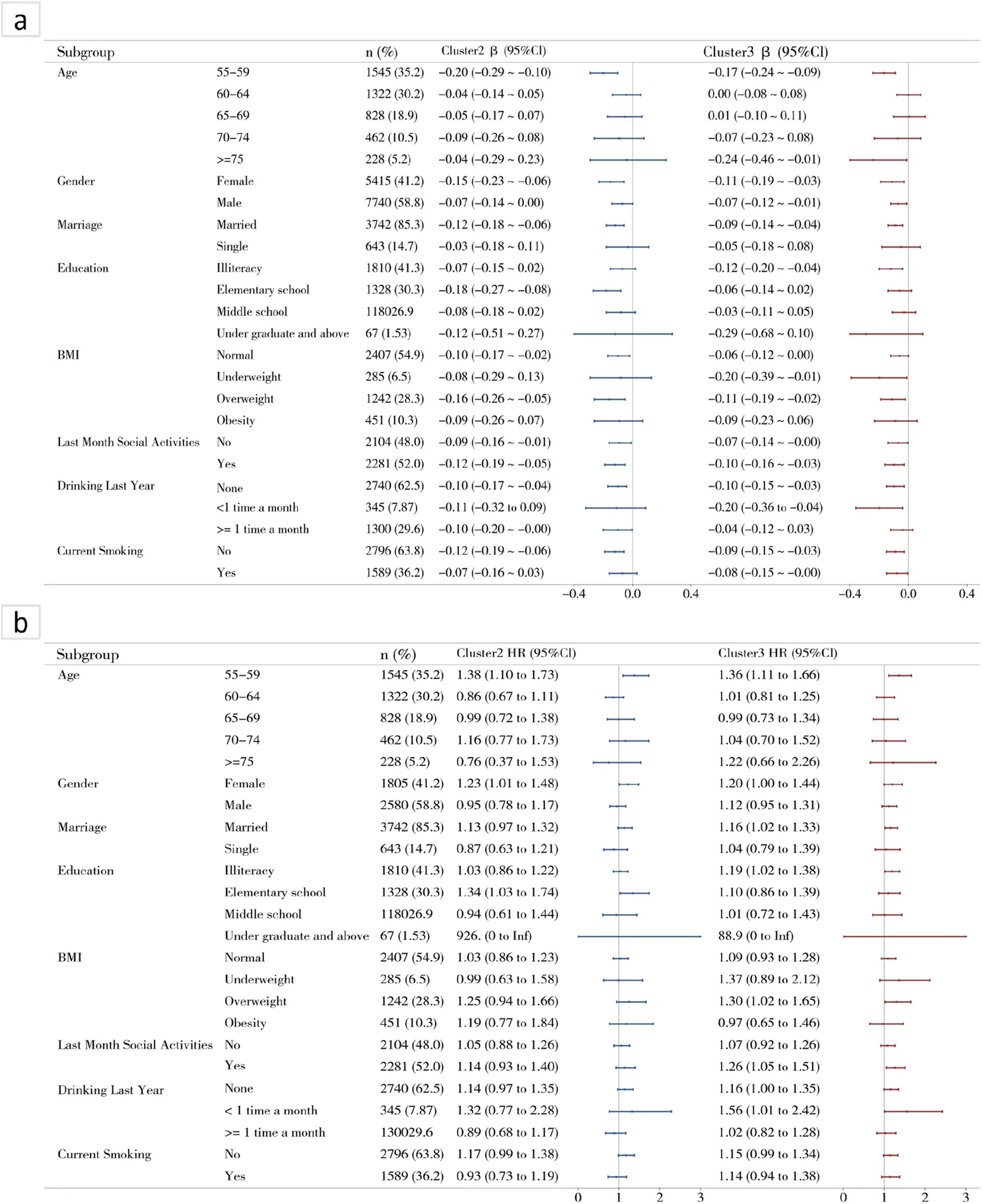
Subgroup analysis results **a** Subgroup analyses on the association of sleep pattern with Cognitive Function Score Decline **b** Subgroup analyses on the association between sleep pattern clusters and AACD-defined cognitive impairment

Figure 3a presents subgroup analyses of the association between sleep pattern clusters and cognitive function decline. Compared with Cluster 1, the strongest associations were observed among younger participants aged 55–59 years (Cluster 2: β = − 0.20; Cluster 3: β = − 0.17) and among participants aged ≥ 75 years for Cluster 3 (β = − 0.24). Females and married individuals showed relatively stronger associations than males or single individuals. Lower educational attainment was associated with larger declines, particularly among participants with elementary schooling (Cluster 2: β = − 0.18) and illiterate participants (Cluster 3: β = − 0.12). For BMI categories, overweight and underweight groups showed relatively larger associations. Socially active participants also demonstrated stronger associations. Lifestyle factors showed generally consistent patterns, with non-drinkers, frequent drinkers, and non-smokers exhibiting significant declines, whereas among current smokers, only Cluster 3 remained statistically significant.

Figure 3b presents subgroup analyses of the association between sleep pattern clusters and AACD-defined cognitive impairment. Compared with Cluster 1, higher likelihoods of AACD-defined cognitive impairment were observed in several subgroups. Notably, Cluster 3 was modestly associated with a higher likelihood among married individuals (HR = 1.16, 95% CI: 1.02–1.33), illiterate participants (HR = 1.19, 95% CI: 1.02–1.38), and overweight individuals (HR = 1.30, 95% CI: 1.02–1.65). Associations were also observed among participants engaged in social activities (HR = 1.26, 95% CI: 1.05–1.51) and those drinking less than once per month (HR = 1.56, 95% CI: 1.01–2.42).

## Discussion

This study, based on the three-wave longitudinal data from CHARLS, using the DTW K-medoids method to identify three distinct sleep patterns. Compared with traditional clustering methods that rely on Euclidean distance, DTW is particularly suitable for longitudinal data because it accounts for temporal misalignments and varying trajectories across individuals [19]. Compared to traditional K-means clustering that relies on means as cluster centers, K-medoids avoids mean bias by using representative samples as cluster centers. By combining DTW with K-medoids, we can obtain more meaningful and longitudinally consistent sleep behavior patterns.

Scholars have attempted to classify sleep patterns using various measurement approaches. Some studies evaluate longitudinal sleep duration based on median sleep time, while others measure sleep duration variability using slope and standard deviation [21]. However, this approach may overlook short-term fluctuations in sleep patterns. Additionally, research has employed K-means clustering to define sleep patterns by integrating multiple sleep indicators such as duration, insomnia, and hypersomnia [22]. This approach is sensitive to initial cluster centers and prone to local optima. We combined longitudinal sleep duration with daytime napping status to identify three sleep patterns using a DTW-based K-medoids approach. Cluster 1 was designated as the control group to investigate changes in cognitive scores and the AACD-defined cognitive impairment.

In models where the outcome variable was cognitive decline, we found that in the final adjusted model, both cluster 2 and cluster 3 exhibited significant cognitive decline, with cluster 2 having a greater impact on cognitive score reduction. In specific cognitive domains, both cluster 2 and cluster 3 individuals showed significant declines in orientation and memory. Only cluster 2 exhibited a significant decline in copying ability. Neither group showed significant declines in attention or calculation. Previous research has shown that sleep duration and cognitive decline share a common threshold: both durations below and above this threshold exhibit a negative correlation with cognitive function, forming a U-shaped relationship. Yanjun Ma et al. found that individuals with insufficient sleep duration or excessive sleep duration exhibited a highly correlated decline in cognitive function. Moreover, a larger β value was observed in individuals with shorter sleep duration. Previous studies have suggested that short sleep duration may be associated with increased amyloid β plaque burden and accelerated ventricular enlargement, which could potentially contribute to neurodegenerative processes related to cognitive decline [7]. However, some studies indicate that a significant risk of cognitive decline is observed only in individuals with prolonged sleep duration, while no significant association between short sleep duration and cognitive function has been found. This discrepancy may stem from the fact that prolonged sleep often accompanies sleep fragmentation. Based on previous research, the relationship between sleep duration and cognitive decline remains controversial. Future studies could further investigate the association between sleep duration and cognitive decline, as well as elucidate the underlying physiological mechanisms.

In the model examining AACD-defined cognitive impairment, the crude model showed that both sleep patterns (Cluster 2 and Cluster 3) were significantly associated with AACD-defined cognitive impairment, and Cluster 2 had a higher hazard ratio. After fully adjusting for covariates, only participants in Cluster 3 showed a modest association with a higher likelihood of AACD-defined cognitive impairment. Although Cluster 2 exhibited a faster rate of decline in continuous cognitive scores, Cluster 3 was modestly associated with a higher likelihood of AACD-defined cognitive impairment. Previous epidemiological studies have reported associations between long sleep duration and cognitive impairment, although findings have not always been entirely consistent. Our findings are consistent with existing research, indicating that prolonged sleep duration may be modestly associated with a higher likelihood of AACD-defined cognitive impairment, whereas this association is not significant among individuals with shorter sleep duration [23–26]. Previous studies have hypothesized that prolonged sleep duration may reflect underlying comorbidities or neuroinflammatory processes associated with poorer cognitive outcomes. Previous neuroimaging studies have reported associations between prolonged sleep duration and structural brain changes, including reduced cortical thickness in specific regions, which may be associated with poorer cognitive outcomes [27].

Subgroup analysis results indicate that younger older adults, women, married individuals, those with lower education levels, and overweight individuals were more likely to exhibit cognitive decline and AACD-defined cognitive impairment. We found that the association between poor sleep patterns and cognitive outcomes was not uniform across different age groups. Notably, the association was most pronounced among individuals aged 55–59, suggesting that sleep disturbances earlier in the aging process may be associated with less favorable cognitive trajectories [28]. We found that unhealthy sleep patterns were associated with lower cognitive scores and a higher likelihood of AACD-defined cognitive impairment among women, which contradicts existing research. For instance, some studies have only identified a significant association between prolonged sleep duration and cognitive decline in men [7]. This discrepancy may stem from the fact that the study relied solely on a single nighttime sleep measurement and may partly reflect sex-related biological differences reported in previous studies in postmenopausal women [29]. Individuals with lower educational attainment demonstrated greater susceptibility, consistent with the cognitive reserve hypothesis [30]. Overweight individuals exhibit reduced cognitive function and higher risk of cognitive impairment, which may be related to metabolic and vascular factors associated with cognitive decline [31]. Even among socially active individuals, unhealthy sleep patterns remain associated with cognitive decline, even when no significant effect is observed—a finding consistent with previous research [32]. Additionally, our findings showed that smoking and drinking behaviors were associated with relatively higher cognitive scores in certain subgroup analyses; however, these findings should be interpreted cautiously due to potential residual confounding and the lack of consistent evidence from previous studies. Future research is needed to further clarify the potential mechanisms underlying the observed associations between sleep trajectory patterns and cognitive impairment in older adults.

## Conclusion

In summary, we identified three longitudinal sleep–napping patterns. Short or long nighttime sleep combined with irregular napping was linked to faster cognitive decline and higher likelihood of AACD-defined cognitive impairment. These results highlight that monitoring sleep trajectories, rather than single measures, can help identify older adults at risk. Interventions promoting stable sleep and moderate, regular napping may help preserve cognition, and integrating sleep monitoring into community health programs could be a cost-effective way to reduce dementia burden.

### Limitations

Several limitations of this study should be acknowledged. First, sleep duration and daytime napping were self-reported, which may be subject to recall bias and measurement error. In addition, objective sleep assessments such as actigraphy, polysomnography, and sleep diaries were unavailable in CHARLS. Second, the cognitive impairment outcome was operationally defined using cognitive score thresholds rather than clinical diagnoses, as standardized clinical assessments such as Mini-Mental State Examination (MMSE), Montreal Cognitive Assessment (MoCA), Clinical Dementia Rating (CDR) were unavailable. Therefore, the findings should be interpreted within the context of an epidemiological, score-based operational definition rather than a clinically confirmed diagnosis. In addition, AACD-defined cognitive impairment was assessed only at survey waves, and therefore the exact timing of onset could not be determined. Third, sleep trajectory patterns were derived from repeated measurements across multiple survey waves; therefore, the temporal relationship between sleep trajectories and cognitive outcomes should be interpreted cautiously.

## Supplementary Information

Supplementary Material 1.

## Data Availability

All original data used in the study from the China Health and Retirement LongitudinalStudy can be freely downloaded from their official websites (https://charls.pku.edu.cn/).

## Abbreviations

MCI: Mild cognitive impairment
CHARLS: China Health and Retirement Longitudinal Study
AACD: Aging-associated cognitive decline
SD: Standard deviation
DTW: Dynamic Time Warping
BMI: Body mass index
HR: Hazard Ratio
