# Supplementary material for: Sleep patterns and impaired cognitive function in adults aged ≥ 55 years: a longitudinal study in China
Source: BMC Public Health. 2026 Jun 2;26:2433. doi: 10.1186/s12889-026-27979-w (PMC13501763; doi:10.1186/s12889-026-27979-w)
Supplement: Supplementary file 1 [file 12889_2026_27979_MOESM1_ESM.docx]

**Supplemental Materials**

**Title:** Sleep Patterns and Impaired Cognitive Function in Adults Aged ≥55 Years: A Longitudinal Study in China

**Authors**: Wei Zhang, DeQing Zeng, Yuge Yan, YiJing Chu, Yuqi Yang, Jing Guo, Jie Shen, Jiaoling Huang

List of contents

[**Ⅰ. Variable Measurement in CHARLS**	1](#_Toc217909769)

[i. Cognitive Score Measurement	1](#_Toc217909770)

[ii. Depression Status Assessment	1](#_Toc217909771)

[**Ⅱ. Supplementary Figure**	2](#_Toc217909772)

[Figure S1 Flow Chart Participants Selected	2](#_Toc217909773)

[**Ⅱ. Supplementary Table**	2](#_Toc217909774)

[Table S1 Intergroup comparisons between different cluster groups*	2](#_Toc217909775)

[Table S2 Longitudinal Association Between Sleep Pattern And MCI	3](#_Toc217909776)

**Appendix**

**Ⅰ. Variable Measurement in CHARLS**

i. Cognitive Score Measurement

The cognitive score measurement in CHARLS involves four dimensions: orientation, memory, attention and calculation, and copying. The questions for orientation include: (1) Please tell me today's date. (2) Please tell me the day of the week. Is it Monday, Tuesday, Wednesday, Thursday, Friday, Saturday, or Sunday? (3) What is the current season (spring, summer, fall, or winter)? The total score is 5 points. The memory dimension involves reading ten words to the subject: “Please listen carefully. I will not repeat them. Try to remember and repeat as many as possible.” After a period, the subject is asked to recall and repeat as many words as possible from what was heard. The maximum score for both word recall attempts is 20 points. Attention and calculation assesses the subject's ability to answer questions like “What is 100 minus 7?” and continue subtracting 7 up to five times, with a maximum score of 5 points. Copying ability is measured by presenting the subject with a diagram of a folded five-pointed star and asking them to draw the image on paper.

ii. Depression Status Assessment

Depressive status was measured using the Center for Epidemiologic Studies Depression Scale-10 (CESD-10). The 10 questions pertained to feelings and behaviors during the past week, with identical response options for each item: (1) Rarely or none of the time (<1 day), (2) Some or a little of the time (1-2 days), (3) Occasionally or a moderate amount of the time (3-4 days), and (4) Most or all of the time (5-7 days). Select the most appropriate response. The total score is out of 30 points. A score of ≥10 indicates the presence of depression. The specific questions are as follows.

1. I was bothered by things that don't usually bother me.

2. I had trouble keeping my mind on what I was doing.

3. I felt depressed.

4. I felt everything I did was an effort

5. I felt hopeful about the future.

6. I felt fearful.

7. My sleep was restless.

8. I was happy.

9. I felt lonely.

10. I could not get "going."

**Ⅱ. Supplementary Figure**

Figure S1 Flow Chart Participants Selected

**Ⅱ. Supplementary Table**

Table S1 Intergroup comparisons between different cluster groups*

|  | **P value adjusted** | | |
| --- | --- | --- | --- |
|  | **Cluster 1 vs Cluster 2** | **Cluster 1 vs Cluster 3** | **Cluster 2 vs Cluster 3** |
| **Age** | <0.01 | 0.99 | <0.001 |
| **gender** | <0.001 | 1.00 | <0.001 |
| **Education** | <0.001 | 0.32 | 0.07 |
| **Drinking Last Year** | <0.001 | 0.84 | <0.01 |
| **Current Smoking** | <0.001 | 0.72 | <0.001 |
| **Heart Attack** | 0.84 | 0.10 | <0.01 |
| **Depression** | <0.001 | <0.001 | <0.001 |

* Continuous variables were analyzed using one-way analysis of variance, while categorical variables were assessed using Pearson's chi-square test.

Table S2 Longitudinal Association Between Sleep Pattern And MCI

|  | **Model 1*** |  | **Model 2**** |  | **Model 3***** |  |
| --- | --- | --- | --- | --- | --- | --- |
|  | **OR (95% CI)** | **p value** | **OR (95% CI)** | **p value** | **OR (95% CI)** | **p value** |
| **Cluster 1** | ref | NA | ref | NA | ref | NA |
| **Cluster 2** | 1.26 (1.10 to 1.44) | <0.001 | 1.12 (0.98 to 1.28) | 0.11 | 1.10 (0.95 to 1.25) | 0.19 |
| **Cluster 3** | 1.16 (1.03 to 1.30) | <0.01 | 1.11 (0.98 to 1.25) | 0.09 | 1.15 (1.02 to 1.23) | <0.01 |

*Model 1 is a crude model of cognitive decline and cluster;

**Model 2 adjusts for age, gender, marriage, education, and BMI;

***Model 3 additionally adjusted for systolic pressure, diastolic pressure, hypertension, diabetes or high blood sugar, heart attack, stroke, depression, last month's social activities, alcohol consumption in the past year, current smoking status and BMI.
